# The whole-genome sequence of the halotolerant nitrogen-fixing bacterium *Azotobacter salinestris* strain AZT-41

**DOI:** 10.1128/mra.00695-25

**Published:** 2025-12-09

**Authors:** Lekhana S. M., Pushya Pradeep, Deepesh Nagarajan, Hanumantharaju K. N., Sreenivasa M. Y., Chennappa Gurikar

**Affiliations:** 1 Department of Food Technology, Faculty of Life and Allied Health Sciences, M. S. Ramaiah University of Applied Sciences 410981 , Bengaluru, Karnataka, India; 2 Department of Biotechnology, Faculty of Life and Allied Health Sciences, M. S. Ramaiah University of Applied Sciences, Bengaluru, Karnataka, India; 3 Department of Studies in Microbiology, University of Mysore, Mysore, Karnataka, India; University of Wisconsin-Madison, Madison, Wisconsin, USA

**Keywords:** *Azotobacter*, WGS, DNA sequencing, *salinestris*, nitrogen fixation

## Abstract

*Azotobacter salinestris*, a diazotrophic, nitrogen-fixing bacterium, promotes plant growth and bioremediation in saline soils. Whole-genome sequencing (5.29 Mb, 30× coverage, *N*_50_: 4840) identified 4,423 genes, including *nif* genes. This study highlights its genomic potential for sustainable agriculture, offering eco-friendly solutions through enhanced nitrogen fixation, soil fertility improvement, and biocontrol applications.

## ANNOUNCEMENT

*Azotobacter salinestris*, a diazotroph, exhibits nitrogen fixation, salinity tolerance, and plant growth-promoting activity via IAA, gibberellin, and ACC deaminase production, particularly under saline stress (1–3). Its antifungal potential has been demonstrated against *Fusarium* species, improving seed germination, vigor index, and seedling growth in cereals (3, 4). *A. salinestris* shows promise in bioremediation by degrading pesticides, solubilizing phosphate and calcite, and enhancing soil fertility in saline and calcareous soils (1, 5, 6). Its effective rhizoplane colonization (7) and stress-mitigation abilities further support its application in sustainable agriculture. The whole-genome sequencing (WGS) of the AZT-41 strain has been presented in this report. This will identify genes for nitrogen fixation, metabolite biosynthesis, and pathogen antagonism, aiding strain improvement and sustainable biocontrol (8, 9).

The strain AZT-41 (formerly GVT-1) of *Azotobacter salinestris* was isolated from soil collected at a 15 cm depth in an agricultural field in Raichur, Karnataka, India (16.275639°N, 76.607962°E) (10). The strain was cultured at 37°C on nitrogen-free Waksman agar for 3–5 days, and genomic DNA was extracted using the QIAamp DNA Blood Mini Kit (Cat# 51104) following the manufacturer’s instructions. The quality of genomic DNA was assessed using Qubit dsDNA High Sensitivity Assay (Invitrogen, Cat# Q32854). The concentration of the DNA was found to be 96.6 ng/µL with a total volume of 40 µL. Agarose gel electrophoresis was used for analyzing the integrity of the DNA.

The isolated DNA was used to generate a DNA library using Roche Kapa HyperPlus Kit (Cat. No. 07962347001). The generated DNA library showed an average size of 346 bp, with the lowest fragment size being 193 bp and the highest fragment size being 721 bp. This was then subjected to sequencing using Illumina NovaSeq 6000 and NovaSeq 6000 S4 Reagent Kit v1.5. A total of 13,311,258 raw reads were obtained, with the mean read length of 151 bp. The raw reads were processed using FastQ mcf (v-1.04.803) (11) to remove adapters and erroneous bases using the following parameters - -q 20x 0.01p 15L 50k 0. *De novo* assembly was performed using SPAdes (v3.11.1) (12) resulting in a genome with GC% of 56.11%. Assembly statistics are summarized in Table 1. The resulting scaffolds were subsequently used for downstream analyses.

**TABLE 1:** *De novo* assembly statistics for sample AZT_41

Parameter	Value
No. of contigs (0 bp)	1
No. of contigs (1,000 bp)	1
No. of contigs (5,000 bp)	1
No. of contigs (10,000 bp)	1
No. of contigs (25,000 bp)	1
No. of contigs (50,000 bp)	1
Largest contig	5,292,277 bp
Total length	5,292,277 bp
*N*_50_	5,292,277 bp
*N*_75_	5,292,277 bp
*L*_50_	1
*L*_75_	1
GC%	56.11

The preliminary assembled genome was further subjected to contamination removal and annotation was performed by NCBI using the Prokaryotic Genome Annotation Pipeline (13) using GenMark-S2+. The annotation performed provided results that included the identification of genes, coding sequences (CDSs), rRNAs, tRNAs, and other non-coding RNAs (ncRNAs). A total of 4,423 genes were predicted, including 4,337 CDSs (4,018 protein-coding), and 86 RNAs comprising six copies each of 5S, 16S, and 23S rRNAs, 64 tRNAs, and 4 ncRNAs. Genomic insights highlight the potential of *A*. *salinestris* in plant growth promotion and sustainable agriculture.

## Data Availability

The genome of *Azotobacter salinestris* strain AZT-41 deposited to under the accession number CP168084.1. The sequence data has been submitted to NCBI-SRA and is available under the accession number SRR31939395. The sequencing project is associated with NCBI Bio Project: PRJNA1102947 and BioSample: SAMN41037731 within GenBank.
